# Implementing effective hygiene promotion: lessons from the process evaluation of an intervention to promote handwashing with soap in rural India

**DOI:** 10.1186/1471-2458-14-1179

**Published:** 2014-11-19

**Authors:** Divya Rajaraman, Kiruba Sankar Varadharajan, Katie Greenland, Val Curtis, Raja Kumar, Wolf-Peter Schmidt, Robert Aunger, Adam Biran

**Affiliations:** Division of Epidemiology and Biostatistics, St John’s Research Institute, St John’s National Academy of Health Sciences, Sarjapur Road, Bangalore, 560034 India; Department of Disease Control, London School of Hygiene & Tropical Medicine, London, WC1 7HT United Kingdom

**Keywords:** Handwashing with soap, Hygiene promotion, Process evaluation, Community-based intervention

## Abstract

**Background:**

An intervention trial of the ‘SuperAmma’ village-level intervention to promote handwashing with soap (HWWS) in rural India demonstrated substantial increases in HWWS amongst the target population. We carried out a process evaluation to assess the implementation of the intervention and the evidence that it had changed the perceived benefits and social norms associated with HWWS. The evaluation also aimed to inform the design of a streamlined shorter intervention and estimate scale up costs.

**Methods:**

Intervention implementation was observed in 7 villages. Semi-structured interviews were conducted with the implementation team, village leaders and representatives of the target population. A questionnaire survey was administered in 174 households in intervention villages and 171 households in control villages to assess exposure to intervention activities, recall of intervention components and evidence that the intervention had produced changes in perceptions that were consistent with the intervention core messages. Costs were estimated for the intervention as delivered, as well as for a hypothetical scale-up to 1,000 villages.

**Results:**

We found that the intervention was largely acceptable to the target population, maintained high fidelity (after some starting problems), and resulted in a high level of exposure to most components. There was a high recall of most intervention activities. Subjects in the intervention villages were more likely than those in control villages to cite reasons for HWWS that were in line with intervention messaging and to believe that HWWS was a social norm. There were no major differences between socio-economic and caste groups in exposure to intervention activities. Reducing the intervention from 4 to 2 contact days, in a scale up scenario, cut the estimated implementation cost from $2,293 to $1,097 per village.

**Conclusions:**

The SuperAmma intervention is capable of achieving good reach across men and women of varied social and economic status, is affordable, and has the potential to be effective at scale, provided that sufficient attention is given to ensuring the quality of intervention delivery.

## Background

Globally, diarrhoea and pneumonia are the leading infectious causes of death in children younger than 5 years. A global review of causes of child mortality in 2010 estimated that 751 000 children died as a result of diarrhoeal disease, while 1.07 million child deaths were attributed to pneumonia. In India alone, it was estimated that there were 212,000 child deaths due to diarrhoea and 397,00 pneumonia-related deaths [1]. Handwashing with soap (HWWS) is a relatively cheap and easy behaviour to adopt, and could potentially halve the incidence of diarrhoeal disease [2] and reduce the risk of acute respiratory infections by 6-44% [3], saving over 600,000 lives a year [4]. Recent years have witnessed a growing body of research to identify determinants and drivers of HWWS in resource constrained settings [5–7] and to assess the feasibility and effectiveness of interventions to improve handwashing behaviour [8–14]. However, several large-scale interventions to promote HWWS in resource-limited settings have met with limited success [12, 15–17], underscoring the need for better evidence and theory to inform programming to promote hygiene behaviour change [18].

In 2011, we conducted a randomized controlled trial to evaluate the efficacy of a village-level intervention to promote HWWS in a rural setting in the southern Indian state of Andhra Pradesh. While there was no active hygiene promotion being conducted in the villages during our study period, our qualitative formative research confirmed that there was high awareness of causal links between dirty hands and disease, although regular handwashing with soap after defecation and before handling food was believed to be infrequent. All households visited during formative research had at least one bar of soap in use. Based on previous research on the drivers of hygiene behaviour [19], we hypothesized that an intervention that focused on non-health messaging would be effective in bringing about behaviour change. We developed an intervention that sought to increase rates of HWWS through messaging that was intended to: i) increase the perceived non-functional benefits of HWWS by linking the practice with the emotional/psychological rewards of good parenting and aspirations for success (nurture and status); ii) increase the perceived costs of not washing hands with soap by making salient the disgusting nature of routine hand contamination (disgust); and, iii) increase social pressure to practice HWWS by creating the impression that it is a normative behaviour - that is, that most people do it and most people believe it should be done (affiliation). Multiple mechanisms for triggering and sustaining behaviour change were thus incorporated within the intervention.

The intervention was designed to be scalable and to be delivered by a small team. It was designed by a professional creative agency (Centre of Gravity) in collaboration with the London School of Hygiene and Tropical Medicine and the St John’s Research Institute. ‘SuperAmma’ was the face of the campaign: a forward-thinking, rural woman who had a loving relationship with her son, taught him good manners, and ensured HWWS amongst family members. An additional comic character was Ladoo Lingam, who had disgusting habits and did not wash his hands with soap. SuperAmma featured in an animated film and both characters were used in street theatre. The intervention aimed to provide opportunities, cues and rewards for repeated practice of HWWS. Components included community events, monitoring of HWWS in schools and households, HWWS report cards and certificates for children, certificates and SuperAmma figures for mothers who pledged to practise HWWS, and visual reminder stickers on front doors and bathroom walls.

The activities and messages were delivered through community events, an event at the *anganwadi* centre (a state run day care centre for pre-school age children), sessions at the village primary school, small group meetings with men and women in the village, and awareness generation activities including a children’s rally, putting up posters around the village and household visits. The theoretical basis and detailed description of the intervention activities and timeline are reported in the trial outcome paper [20]. A full description of the intervention, intervention materials and short films about the intervention are available on the SuperAmma website (http://www.superamma.org).

The results of the trial showed that HWWS at key occasions was uniformly low (1-2%) at base line and increased to 19% in intervention villages 6 weeks after the intervention, compared to 4% in control villages [20]. At 6 weeks post-intervention there was substantial variation in the outcome across the intervention villages with HWWS ranging from 5-61%. This variation reduced over time. At 6 months post-intervention HHWS at key occasions was 37% in the intervention arm compared with 6% in the control arm. The increase being almost entirely due to continued increases in HWWS in those intervention villages that had shown low prevalence of HWWS in the first follow up. At 12 months post intervention, HWWS at key times stood at 29%. A modified version of the intervention comprising fewer implementation visits was delivered to the original control villages, increasing HWWS at key times to 29% in these villages as well.

Process evaluations of public health interventions can help to interpret the results of a trial through assessing quality, fidelity, reach and receipt of intervention delivery, strengthen future intervention design, and improve cost-effectiveness [21, 22]. In this paper, we report the findings of a mixed methods process evaluation which we conducted to explore the acceptability of the intervention, and to assess the fidelity of delivery and the extent to which the intervention had reached the target population and changed perceptions about HWWS. We also used the findings to inform the design of the short version of the intervention, and we estimated the costs of the long and short versions to inform discussions about scalability.

## Methods

### Data sources and analysis

#### Observation of implementation

In all intervention villages 3 fieldworkers observed the delivery of all activities and recorded whether or not scheduled activities were implemented in a manner that was aligned with the intervention manual. The fieldworkers were experienced in data collection and were independent from the intervention delivery team. They received training in the intervention schedule and components, and were instructed not to intervene with it. They measured attendance at community events by a head count. The quantitative observation data were entered into a spreadsheet. The fieldworkers also wrote qualitative descriptions of the activities they observed, noting the manner in which they were implemented, any problems in delivery, and any changes to the planned order of execution. Two of the study authors (DR and KSV) attended all intervention events and took detailed qualitative field notes on the quality of implementation in the second and sixth villages to receive the intervention. The qualitative data were manually coded under the general themes of acceptability (things liked and not liked), feasibility (barriers and facilitators), impact (positive and negative), and suggestions for improvement.

#### Semi-structured interviews

In the second and sixth villages, semi-structured interviews were conducted with 7 key informants (village leaders, school principals, a teacher, and *anganwadi* workers) and 24 respondents from the target population (11 women who had taken the HWWS pledge, 8 women who had not taken the pledge and 5 men). The 3 members of the implementation team were also interviewed.

All interviews were conducted in the local language, Telugu, through an English speaking translator and were digitally recorded and transcribed in English. The transcripts were reviewed by the interviewer for accuracy and were analysed by the first author using NVivo software. Analysis was thematic by intervention component, and under the general themes of acceptability feasibility, impact, and suggestions for improvement.

#### Questionnaire survey

A verbally administered questionnaire survey assessed exposure to intervention activities, recall of intervention components and messages, and perceptions of handwashing in relation to core intervention messages (reasons for HWWS and perceived norms for HWWS). The survey was carried out in the same households (25 per village) that had been selected and recruited for the collection of handwashing outcome data for the main trial. A socio-economic questionnaire had been administered to these households at the time of recruitment [20]. Respondents were women who were over 16 years of age. The survey was administered 4 to 6 weeks after the completion of intervention activities by the same 3 fieldworkers who had observed the implementation of the intervention.

Questionnaire survey data were entered into an SPSS database [23]. Bivariate analysis was conducted to assess i) self-reported exposure to activities and messaging in intervention and control villages; ii) differences in reported reasons for HWWS and social norms with regard to HWWS in intervention and control villages; and, iii) associations between demographic and socio-economic characteristics, and self-reported exposure to intervention messages and mediators.

#### Intervention costs

We report the actual costs of creating the intervention (excluding research team time and travel), the estimated costs of delivering the long version in the trial villages, and the indicative costs of implementing the short version in an operational context to 1,000 rural villages of similar size (population of 700–2,000) in Andhra Pradesh. All costs are reported in US dollars. The actual costs of delivery are estimated, since production and delivery were sub-contracted to the creative agency and implementation agency, and itemized receipts for all cost components were not available to the research team.

### Ethics

The study protocol received ethical approval from the St John’s Medical College Institution Ethics Committee and the London School of Hygiene & Tropical Medicine Institutional Ethical Review Board. All interviewed respondents provided written informed consent for participation in the study.

## Results

### Acceptability of the intervention

Key informants and members of the target population were generally positive in their opinions about the intervention. They endorsed the goals and found the films and skits enjoyable. Women in particular related to the SuperAmma character and felt that she was a good role model for mothers. Perceptions of the intervention team were also favourable, being viewed as polite and entertaining. In the words of one of the school principals:

“[the intervention team] cooperated completely with us, and made the programme very successful. They said, ‘sir, we will come on this day, at this time, and do this activity – you check if the hands are washed…’ They also conducted the programme well.”

Some villagers expressed curiosity about who was behind the intervention: although they had been informed that it was an NGO working in partnership with a local hospital, a few people speculated that a soap company could be sponsoring the intervention or a politician might be using it as a vehicle for future electioneering.

Respondents who had taken the pledge felt that it brought greater commitment to behaviour change. The volunteer from one of the villages spoke about the importance of the activity, while recognizing that not everyone took the pledge:

“[The pledge] is 100% required, as we tend to forget. If I tell you that I will come somewhere, then even if there is rain or wind, I will still come. To keep up our word, we take a pledge…Some of them did not take the pledge [with the motion of] stretching out their hands, but even if their inner consciousness was aligned, it is enough”.

Not all aspects of the intervention were universally acceptable. For example, there were mixed opinions with regard to the HWWS pledge. Many women and men in the villages showed reluctance and lack of enthusiasm in pledging, even when they recognized the benefits. Members of the Muslim community in particular were concerned that taking a public pledge might contravene their religious beliefs. The intervention promoters felt that the language for the pledge was too long, resulting in wavering attention and lack of sincerity amongst those who agreed to take the pledge.

Observation and interviews suggested that Ladoo Lingam, the comical character who did not wash his hands in the skit, was perceived to be too disgusting in his habits by adult women in the early intervention villages. This character was consequently toned down and subsequent interviews with villagers indicated that the combination of disgust at dirty hands and amusement worked well to increase the memorability of the intervention activities and messages.

While the school activities were generally welcomed by the school management (permission had been sought from the District Education Office before the intervention), the acceptability of the children’s rally was questioned by a couple of school principals in light of the potential safety risk of children walking through the streets.

The children appeared to enjoy the intervention activities and participated enthusiastically, although intervention observation indicated that the promoters could be didactic and did not use all opportunities to encourage active participation. Compared to the children, there was more variation in active participation amongst adults across events and activities, with some villagers showing great interest and amusement and others appearing indifferent to the messages. Also, while all village leaders endorsed the intervention, some played a more active role in promoting the messages through their engagement with the audiences during community events. In one village, a lawyer who was a prominent personality went door-to-door to promote HWWS, which may have created greater community support for the intervention. In contrast, the volunteer in another village was a young woman who lacked confidence and believed that she was not welcomed by many of the village households because she belonged to a lower caste. The enthusiasm and ability of the volunteer to engage the villagers during daily household visits varied between villages, depending on age, social status, knowledge and personality. However, there was no significant association between strength of the village volunteer (qualitatively rated) and the village level change in HWWS.

### Implementation fidelity

Implementation fidelity (delivery as planned) was good overall. The major activities (meetings with village leaders, community events, school events, *anganwadi* centre events, school rallies, monitoring HWWS at the mid-day meal at schools) were delivered in all villages.

At the start of the intervention, a few components were omitted or implemented inconsistently in the first three villages. Posters were not put up in the indicated quantity, the truck was not driven through the village playing the jingle and the intervention team did not gather men for group meetings in the evenings. There were also technical problems. The generator, sound system, and laptop on which the animated films were screened all encountered some malfunctioning and the truck experienced mechanical problems. The intervention team addressed these issues and the intervention progressed with no major technical problems from the fourth village onwards.

A few components of the intervention remained difficult to execute in all villages. One was the ‘common board’ listing the names of all villagers who had taken the HWWS pledge, which was meant to be displayed in a public area. In some villages, it was difficult to find a secure public area and the common board kept falling, was put away or even stolen. A second challenge was printing localised intervention posters with photos of village leaders endorsing HWWS. The printer that was purchased for the intervention was difficult to handle in field conditions and the printing of these posters was only possible when the intervention team could access a print shop in a nearby town.

While supervised HWWS was supposed to be practiced on 4 days in every village school, it was missed on at least one day in 6 of the 7 villages, because of school closures due to holidays, weather or teachers’ meetings. A couple of teachers also expressed reservations about the feasibility of implementing HWWS regularly in schools on account of water consumption, as none of the schools had piped water. The HWWS school report cards proved unsuitable for the youngest students who were not able to follow the instructions.

### Reach and self-reported exposure

On the first day of the intervention, the handwashing promoters made door-to-door visits to all households in the village to issue an invitation to the community event. The headcount of participants at the community events indicated that 17% - 37% of the total village population attended on Day 1, 14% - 28% on Day 2, and 13% - 29% on Day 4 (we are not able to say how many of the same people attended the community events across the 3 days). Participation was highest on all three days in the village with the greatest change in HWWS (Village 7, 61% increase in HWWS at key times). However, there was no clear relationship between village participation in the community events and observed change in HWWS across the villages. Although the intervention was targeted towards women and school-going children, a substantial number of adult men also attended the events in some villages (see Table 1).

**Table 1:** Reach of community events,* retention of intervention paraphernalia, and change in HWWS

% of population	Village							
	1	2	3	4	5	6	7	Total
Average Women’s Attendance	34	22	27	39	28	25	41	31
Average Men’s Attendance	22	14	13	22	23	17	30	20
Average Children’s Attendance	23	12	21	23	12	20	23	19
Average Total Attendance	27	16	20	28	21	21	31	23
Respondents who reported taking the HWWS pledge	96	96	88	88	92	64	60	83
Respondents with Super Amma certificate	96	83	84	84	84	60	56	78
Households where Super Amma certificate was on display	12	0	12	4	16	8	8	9
Respondents who received Super Amma figurine after pledge	96	79	84	84	84	60.0	56	78
Households with a HWWS door sticker or poster	33	17	58	44	20	40	36	36
Households with a HWWS sticker in bath room	20	25	21	24	0	24	12	18
*% Change in HWWS on key occasions*	*7*	*5*	*9*	*7*	*16*	*22*	*43*	*18*
*% Change in HWWS on all occasions*	*5*	*5*	*8*	*5*	*30*	*19*	*61*	*16*

*Average of the attendance recorded on 3 days. Attendance was calculated as a proportion: number of people who attended the event out of the number of people residing in the village, as recorded in a census taken at the start of the study.

In line with observations, the questionnaire survey found that self-reported exposure to intervention activities was high amongst the target population, with over 80% of respondents recalling the animated films, skit, children’s rally, posters, household visits, the intervention truck and the children’s HWWS report card. There were no major differences between intervention villages in self-reported exposure to these intervention elements (data not shown). However, the proportion of respondents who had taken the HWWS pledge, while being above 80% overall, showed significant variation at the village level; a similar pattern was observed in the proportion of women who reported that they had received a SuperAmma certificate or figurine during pledging, and who received a SuperAmma gift soap for taking the pledge on the final day of the intervention. The retention of the SuperAmma certificate among recipients was high, at about 79% of surveyed households in all villages 4–6 weeks after the intervention. On average, 36% of the households had a SuperAmma door sticker or poster displayed, and 18% had a SuperAmma sticker in their bathroom area, although there was substantial variation between villages (Table 1).

We carried out sub-group analyses to explore whether there were any variations in exposure to the intervention and participation in the pledging exercise across different social and economic groups (Table 2). Exposure to most intervention activities was high (over 70%) across socio-economic groups, with no statistically significant differences between income and occupational groups. The only activity which showed a statistically significant difference between groups was participation in pledging: Hindus were more likely to have taken the pledge compared to Muslims (85% versus 56%, p < 0.01), and women in landowning households were somewhat less likely to have taken the pledge than those in households with no land (78% versus 96%, p < 0.05).

**Table 2:** **Socio-economic variables and exposure to/participation in intervention activities in intervention villages**

	Educational status			Occupation			Religion/Caste			Land ownership		
	No education (n = 80)	Primary education (n = 77)	Secondary or higher (n = 16)	Agricultural or other labour (n = 143)	Housewife (n = 15)	Others (n = 11)	Backward and forward castes (n = 119)	Scheduled castes and tribes (n = 43)	Muslims (n = 11)	No lands (n = 52)	Up to 2.5 acres of land (n = 96)	More than 2.5 acres of land (n = 27)
**Self-reported exposure to intervention components**												
Seen an intervention about HWWS	84	70	88	78	79	82	77	81	73	83	76	77
Seen SuperAmma film	85	81	94	85	74	91	85	81	82	88	83	77
Seen skit	87	71	94	82	63	91	82	79	73	90	77	77
Seen HWWS posters with village headman	90	91	100	92	84	100	96	84	73	98	88	89
Seen intervention truck	97	96	100	96	100	100	97	95	100	100	97	92
Heard SuperAmma intervention jingle	98	95	100	96	100	100	98	93	100	96	98	92
**Participation in intervention activities**												
Took the HWWS pledge	86	81	88	85	68	100	85	88	**55** ^**1**^	**96** ^**2**^	81	69
HWWS door sticker or poster at home	32	38	44	37	26	36	37	35	27	22	38	54

^1^p <0.05, compared to Hindus.

^2^p < 0.01, compared to landowners.

### Perceptions regarding HWWS practices

The post-implementation questionnaire survey showed that adult women in the intervention villages were considerably more likely than those in control villages to be aware of intervention-related HWWS messages, with all differences receiving strong statistical support (p < 0.05) (Table 3). For example, 95% of respondents in the intervention villages identified ‘after defecation’ as well as ‘before eating’ as important occasions for HWWS, compared to 12% and 29% of respondents in control villages. In open ended questions about why it was important to wash hands with soap, respondents in intervention villages were also much more likely than those in control villages to cite reasons that were in line with intervention messages. These included good manners (84% versus 21%), to be successful (30% versus 0%) and to protect children (63% versus 2%). Prevention of disease was the most frequently cited reason for HWWS in both the intervention and control villages with respondents from intervention villages more likely to cite health benefits than those in control villages (99% versus 48%).

**Table 3:** **Reported occasions, reasons and social norms for HWWS in intervention and control villages**

			Intervention villages					
			Education			Caste/Religion		
	Intervention villages N = 174	Control villages N = 171	None (80)	Primary/secondary incomplete (77)	Secondary or higher (16)	Higher caste (119)	Scheduled caste or tribe (43)	Muslims (11)
**Perceived importance of HWWS**								
After defecation	95	12	94	96	100	94	98	100
Before eating	95	29	93	97	94	96	91	100
Before cooking	89	7	84	94	94	89	86	100
**Reasons for HWWS**								
To be healthy/prevent disease	~100	48	99	100	100	100	98	100
To be successful	30	0	21	35	44	33	26	9
To have good manners	84	21	78	91	81	87	77	82
To protect our children	63	2	61	62	75	61	70	55
That is what everyone does here	8	0	6	8	13	5	14	9
**Norms about HWWS**								
Almost everyone in village washes hands with soap after defecation	35	8	36	31	44	36	31	44
Almost everyone in village washes hands with soap before eating	36	10	34	40	31	34	40	31

Normative beliefs about HWWS in the community were different in intervention and control villages. Respondents in the intervention villages were more likely to report that ‘almost everyone in this village washes hands with soap after defecation’ (35% versus 8%, p < 0.05) and that ‘almost everyone in this village washes hands with soap before eating’ (36% versus 10%, p < 0.05). Respondents from intervention villages were also more likely to report that people in their village wash hands with soap more than people in other villages in the area (98% versus 42%, p < 0.05). Bivariate and multivariate models to assess associations of these perceptions with changes in household handwashing rates did not yield any significant associations.

The results of the questionnaire survey were corroborated by qualitative data indicating that villagers who had been exposed to the intervention understood the key messages that HWWS should be undertaken after defecation and before eating. Respondents also mentioned that parents should follow good habits of HWWS and set an example for children (manners and nurture message), that clean habits lead to success in life (success message) and that hands get contaminated by daily activities (disgust message). Although the intervention had not explicitly addressed health, many also spoke about the health benefits of HWWS.

### Intervention cost

#### Development

The costs of developing the intervention amounted to a total of $79,288. This includes $39,854 for the development of the animated ‘Two Hands Film’ and initial storyboards by a British Agency (Good Pilot), and $39,354 for the development of all other intervention activities and materials by an Indian Agency (Centre of Gravity) as described in our earlier paper [20]. The development costs represent a one-off investment, and do not include research team staff time and travel costs. These costs would not be incurred in a scaling up/replication of the intervention.

#### Implementation

The delivery of the long intervention in seven villages cost a total of $2,293 per village (details shown in Table 4). The villages ranged in population size from 773 to 1457 people. After salary and accommodation costs for the intervention team, the greatest expense was for equipment: the generator, audio system, projector and two vehicles that were hired for the month in which the intervention was delivered. The cost of consumables was $559 per village (mainly materials such as posters, stickers, SuperAmma figurines, and certificates).

**Table 4:** **Long intervention estimated delivery costs and short intervention projected scale up costs (US Dollars)**

	Long intervention (7 villages)	Short intervention (1,000 villages)^1^
*Equipment*		
Purchased equipment	2,425	60,206
Hired equipment	4,378	158,460
*Intervention Materials*		
Non-consumables^2^	356	3,561
Consumables	3,915	559,288
Human Resources		
Staff hire	4,978	315,638
10 teams for 10 months		
***Total Cost***	*16,053*	*1,097,153*
***Cost Per Village***	*2,293*	*1,097*

^1^Scale-up costs are based on cost of equipping a team (with equipment and intervention materials that are reused in each village) and assume that 10 teams deliver the intervention over 10 months in 100 villages (total of 1000 villages reached over one calendar year). Per team staff costs include two promoters, one supervisor and a driver. All equipment would be purchased except for the intervention truck and vehicle for each project team (this cost includes fuel for the vehicles and generator). Costs are for delivery in similar-sized villages in Telugu language (500–2,000 population).

^2^Non-consumables such as flip-charts, uniforms for implementers, and a model clock were produced just once for use throughout the intervention.

As an indication of the financial implications of taking the intervention to scale, we estimated the cost of delivering the short intervention to 1,000 villages in Andhra Pradesh (where there would be no need for language translation). The calculation assumes that the intervention is delivered over one calendar year by a total of 10 teams, each working five-day weeks and travelling for one day per week. It factors in two months of school holidays during which the intervention cannot be delivered. We assume that we could dispense with the technical assistant by training the driver of each team and compensating them for providing technical and logistical support. As implementation would take place over one year, some equipment that was previously hired (audio system, projector, generator) would be purchased. We estimate the cost at $1,097 per village (Table 4).

## Discussion

We found that the intervention was implemented largely as intended (albeit with some variation in quality) and was well received overall by adults and children. We also found differences between intervention and non-intervention villages in reported reasons for HWWS and perceived norms of handwashing practices. Taken together, these two pieces of evidence strengthen confidence in the conclusion that the increased rates of HWWS in intervention villages reported previously [20] were due to the effect of the intervention.

The process evaluation provides a possible explanation for the substantial inter-village variation in the impact of the intervention by showing that the quality of the intervention was lower in the first three villages in which it was delivered, which is also where the observed change in HWWS was lowest. This highlights the potential vulnerability of a short duration intervention to fluctuation in quality. Whilst a large-scale intervention programme might see sequential improvements as implementers gain skill, it might also see a fall off in quality due to poor supervision or motivational fatigue in key staff.

Recent research on hygiene promotion from Nepal suggests that social marketing interventions may be effective in improving population level health outcomes, but not reach the ultra-poor [24]. However, in our study, the lack of difference in exposure to most intervention components across social and economic strata was encouraging, demonstrating the ability of community events and household visits to cut across the strong social divides that exist within most Indian villages. The only marked difference was that Muslims were less likely to take the HWWS pledge, although they were still willing to listen and watch. This finding points to the importance of pilot testing to ensure the acceptability and delivery strategy for the pledging activity, should the intervention be transferred to a different cultural setting.

Although the intervention was primarily targeted towards mothers and children, male attendance at community events was similar to that of women (male exposure to other elements of the intervention was not assessed). Changes in male HWWS practices were reported to be of a similar magnitude to those observed in women [20]. The results suggest that the materials also resonated with men and that their involvement is probably beneficial to the overall impact of the intervention. It is possible that this impact could be increased in the future by developing intervention components that specifically target the adult male population.

Our survey detected differences in HWWS mediating factors that could plausibly be attributed to the intervention, particularly an association with good manners and an increase in the extent to which HWWS was perceived as normative. However, even in intervention villages, only a minority of respondents thought that most people practice HWWS after defecation or before eating. It is also possible that practicing HWWS makes respondents more likely to perceive and/or report the behaviour as a social norm rather than vice versa.

It is striking that the most commonly cited benefit of HWWS amongst respondents to the household survey was to protect health, even though the intervention did not include explicit health messaging. This was true in both the control and intervention villages. This finding supports the formative research conclusion that the target population was already aware about the importance of HWWS for health, but that this health knowledge alone had been insufficient to produce widespread adoption of HWWS. It is interesting that respondents from intervention villages cited the health benefits of HWWS more often than in the non-intervention villages. It is possible that latent knowledge of the health benefits of HWWS was brought to the fore by exposure to the intervention or that the messages about disgust made non-specific health benefits of HWWS more salient. If this is so, it may be that the success of the intervention was in part due to the underlying awareness of health benefits. It may also be that respondents are more likely to give a rational and socially acceptable explanation for their behaviour in a structured interview setting even if their behaviour were strongly influenced by non-functional benefits. We cannot comment on the extent to which the intervention results would generalise to a population that lacked any prior exposure to health messages. However, our experience of formative research for handwashing promotion across a wide variety of countries leads us to believe that such populations are rare.

The process evaluation provided valuable information for designing a shorter version of the intervention that would be less expensive to implement at scale. Lessons learned included the need for greater engagement of village leaders and a more participatory approach with children. Some of the contact time was reduced, as was repetition of activities such as the community event and *anganwadi* centre event, which reached the same audience several times. The school rally and the truck driving through small village roads playing music were taken out because of safety concerns, the HWWS report cards were restricted to older students because younger students did not understand it, and the common board was omitted because it had proved difficult to implement.

The short format reduced the costs of delivery by about half, to approximately $1,097 per village. These costs could potentially be further reduced by engaging mass media, though it is not clear how effective mass media is when compared with intensive community engagement [16, 17]. A study of handwashing in Ghana found that mass media was less effective per contact reached, but more cost-effective than community engagement [13]. A further challenge to the effectiveness of HWWS interventions in resource limited settings may be the availability of water and soap in schools [8, 25], which can be a constraint to establishing HWWS routines for children through the day.

A limitation of this study is our inability to separate the individual contribution made by the different activities to the overall effect of the intervention, or to assess the importance of underlying knowledge about health benefits of HWWS for bringing about behaviour change. We also lack validated tools for assessing intervening psychological mediating variables for HWWS behaviour change and our study was not designed to test the theoretical basis of the intervention. Our survey may not have fully captured these mediators, but only provided an indication of whether differences in perceptions about HWWS between intervention and control groups were in line with the central messages of the intervention.

## Conclusion

The SuperAmma intervention, which employed messages of disgust, social aspiration, nurture and norms, appears to have been successful in changing handwashing behaviour and sustaining that change up to a year post intervention [20]. The process evaluation showed that the intervention achieved good fidelity, acceptability and reach across men and women of varied social and economic status, and a shorter, equally effective version of the intervention could be delivered at an estimated cost of $1,097 per village. Application of the intervention in a different cultural setting and environment would need to be preceded by pilot research to confirm the acceptability and feasibility of key elements across population groups. The effectiveness of the intervention is likely to be enhanced by parallel efforts to ensure continuous access to soap and water in school and home settings, and may benefit from activities that are specifically targeted to include men.
